# Agro-Climatic Information to Enhance the Machine-Learning Classification of Olive Oils from Near-Infrared Spectra

**DOI:** 10.1021/acsagscitech.4c00355

**Published:** 2024-10-11

**Authors:** María Isabel Sánchez-Rodríguez, Elena Sánchez-López, Alberto Marinas, José María Caridad, Francisco José Urbano

**Affiliations:** † Department of Statistics and Business, Faculty of Law and Business, University of Cordoba, Avda. Puerta Nueva, s/n, 14071 Cordoba, Spain; ‡ Department of Organic Chemistry, University of Cordoba, Campus de Rabanales, Marie Curie Building, 14014 Cordoba, Spain; § Department of Organic Chemistry, University of Cordoba, Campus de Rabanales, Marie Curie Building, 14014 Cordoba, Spain; ∥ Department of Statistics and Business, Faculty of Law and Business, University of Cordoba, Avda. Puerta Nueva, s/n, 14071 Cordoba, Spain; ⊥ Department of Organic Chemistry, University of Cordoba, Campus de Rabanales, Marie Curie Building, 14014 Cordoba, Spain

**Keywords:** extra virgin olive oil, quality and authenticity, near-infrared spectroscopy, agro-climatic data, principal component analysis, machine-learning classification

## Abstract

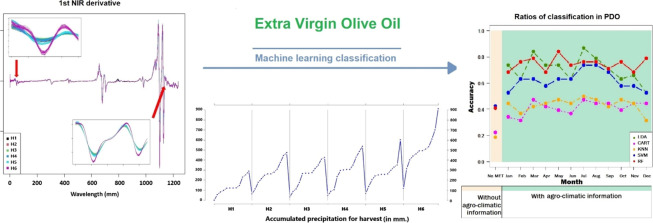

The integrity of extra virgin olive oil (EVOO) quality markers can be compromised owing to deceptive marketing practices, such as misleading geographical origin claims or counterfeit certification labels, i.e., protected designations of origin (PDO). Therefore, it is imperative to introduce ecofriendly, rapid, and economical analytical methods for authenticating EVOO, such as near-infrared (NIR) spectroscopy. Unlike traditional techniques such as chromatography, NIR spectra contain unresolved bands; hence, chemometric tools such as principal component analysis (PCA) are essential for extracting valuable information from them. Herein, PCA was employed to reduce the high dimensionality of the NIR spectra. The PCA factors were then integrated as explanatory variables in machine-learning classification models, enabling the classification of EVOO based on its geographical origin or PDO. Furthermore, the classification models were improved by incorporating agro-climatic data, resulting in a noticeable improvement in the accuracy and reliability of the results. These results were cross-validated by changing the calibration and validation subsamples in successive iterations and averaging the obtained ratios. The results were robust when the olive varieties differed. Consequently, our findings highlight the potential benefits of incorporating agro-climatic information with NIR spectral data in classification models.

## 1 Introduction

Extra virgin olive oil (EVOO) is an important component in the Mediterranean diet, with Spain reigning as the foremost global producer and Andalusia accounting for 80% of national production. EVOO is widely valued for its exquisite flavor and health-enhancing attributes,^1,2^ and its production strictly adheres to mechanical processes to preserve its desirable characteristics. The integrity of EVOO quality indices faces potential threats in the market, including adulteration with less expensive oils or false claims about its geographical origin. Thus, it is imperative to trace precise geographical origins to safeguard the authenticity of olive oil standards. Furthermore, the European Union mandates two distinct certification labels: protected designations of origin (PDO) and protected geographical indications.^3^

Establishing reliable authentication procedures for oils is important in this context. Although classical analytical techniques (i.e., gas or liquid chromatography) offer well-resolved information and are commonly used for olive oil traceability analysis,^4−6^ they are expensive, slow, and involve sample manipulation and solvent use. Conversely, vibrational spectroscopic analytical methods such as near-infrared (NIR), mid-infrared (MIR), or Raman provide a rapid and cost-efficient means to capture a comprehensive chemical profile of oils. However, these techniques present overlapping bands that are less resolved than conventional procedures. Furthermore, the application of mathematical and statistical methods to analyze and interpret data (chemometrics) is necessary to obtain information for EVOO authentication.^7−13^

Principal component analysis (PCA) is commonly employed to provide high-dimensional information in statistical models, particularly when there are more explanatory variables than observations, resulting in deviations from the typical assumptions of the general linear model. By maximizing the variability, linear combinations of independent variables are determined using PCA. These PCA factors can then be incorporated as predictor variables in cause–effect models to predict a dependent (quantitative or qualitative) variable. In this study, each case (oil) was assigned an NIR spectrum consisting of 1237 measurements (which captured the amount of energy absorbed at 1237 wavelengths). PCA was used to summarize the information from the 1237 measurements into a few components, or explanatory variables. Other studies have also utilized PCA to summarize NIR data for predicting chemical compounds in EVOO^7,14,15^ or for classifying oils using machine-learning algorithms such as linear discriminant analysis (LDA), support vector machines (SVMs), or random forests (RFs).^10,16−18^ Presently, the term *machine learning* encompasses procedures that use statistical techniques, artificial intelligence, and/or computational science to identify patterns and make data-driven decisions. Further details about PCA and the machine-learning procedures that are commonly used in oil classification are included and described in the methodology section (Section 2.2).

Numerous studies in the literature have examined the effect of agro-climatic or meteorological conditions on various aspects of EVOO, specifically focusing on its components.^19−22^ However, few studies have considered that agro-climatic information can be combined with NIR spectral data to predict qualitative features of EVOO, such as geographical origin or PDO. Additionally, in the context of multivariate statistical techniques, the aforementioned studies incorporated agro-climatic information as a non-numerical variable or factor. Typically, this factor is employed as an independent variable in an analysis of variance (ANOVA) or multivariate analysis of variance model to identify groups that are then used in a nonparametric Kruskal–Wallis test or as an LDA output. For example, Damak et al.^19^ used the Kruskal–Wallis test to relate the multielemental composition of olive oils with the climatic characteristics of Tunisia. Mafrica et al.^20^ studied the effects of climate on olive oil quality using ANOVA to identify relevant variations in the chemical parameters of monovarietal olive oils between two variables: growing environment and harvesting time. However, our study takes a different approach: rather than grouping agro-climatic data into factors, we used all the original daily data. In particular, this study considers the comprehensive agro-climatic database sourced from the official webpage of the Automatic Weather Stations (AWSs) in Andalusia. Specifically, the following long-term daily data spanning six harvests were procured: temperature (*Temp*), humidity (*Hum*), wind speed (*WSpe*) and direction (*WDir*), radiation (*Rad*), precipitation (*Precip*), and evapotranspiration (*ETo*).

Previous studies^23−25^ explored the use of agro-climatic information in addition to spectral data to predict a quantitative variable of EVOO (mainly its fatty acid profile). However, the application of meteorological information in machine-learning classification (i.e., estimating an EVOO qualitative variable, such as geographical origin or PDO) is a novel concept. Therefore, the goal of this study was to first obtain the high-dimensional information present in EVOO NIR spectra using PCA analysis, and then use the corresponding PCA factors as input variables in some machine-learning classification models to predict the geographical origin and PDO of the EVOO. Furthermore, agro-climatic information was added as explanatory variables in the classification models. When both NIR and meteorological data were included, the calibration and validation results improved (i.e., the estimation errors calculated from the data included or not in the modeling). Therefore, the potential of agro-climatic information to improve machine-learning classification models is highlighted, with humidity being the variable that produced the greatest improvement. These results were cross-validated i.e., the calibration and validation subsamples were changed in successive iterations and the ratios obtained in each case were averaged. In addition, the robustness of the results was emphasized because the correct classification ratios were consistent when the olive variety varied. Overall, this study is novel because it incorporates agro-climatic data with NIR spectral data to improve the classification and authentication of EVOO.

The paper is structured as follows. Section 2 outlines the procedure for obtaining NIR and agro-climatic data, along with the methodology employed. Section 3 describes the motivation for adding agro-climatic information to NIR spectral data in statistical models, as well as the calibration and validation results for machine-learning classification based on geographical origin and PDO. Finally, Section 4 presents the principal outcomes of the paper.

## 2 Materials and Methods

### 2.1 Data

#### 2.1.1 NIR Data

Two hundred and twenty-two different EVOOs were analyzed from six consecutive harvests: H1 (2005-06) to H6 (2010-11). The geographical origin of the EVOO was one of the following Andalusian provinces: “Cádiz,” “Córdoba,” “Granada,” “Jaén,” “Málaga,” or “Sevilla.” In addition, each EVOO was associated with one of the subsequent PDOs: “Antequera,” “Campiña de Jaén,” “Estepa,” “Lucena,” “Montoro–Adamuz,” “Poniente de Granada,” “Priego Córdoba,” and “Sierra Cádiz.” In regards to the olive variety, the majority of the analyzed oils were from “Hojiblanca” (29.8%) and “Picual” (34.7%) olives. The frequency distributions of these variables are shown in Table A.1 (Appendix A).

Chemical data from the EVOO samples were obtained using NIR spectroscopy. The data acquisition process entailed a two-phase centrifugation system and utilized a Spectrum One Near-Infrared Testing System (NTS) Fourier transform (FT)–NIR spectrophotometer (PerkinElmer LLC, Shelton) provided by an integrating sphere module. A glass Petri dish and a hexagonal reflector with a path length of ∼0.5 mm were used for transreflectance analysis. The radiation was reflected by a diffuse stainless-steel surface, which directed it back through the sample to the reflectance detector. The spectral data were acquired using Spectrum Software version 5.0.1 to ensure precision and reduce measurement inconsistencies. To improve the data quality, two distinct reflectors were employed, and the resulting averaged outcomes were used.

**Figure 1:**
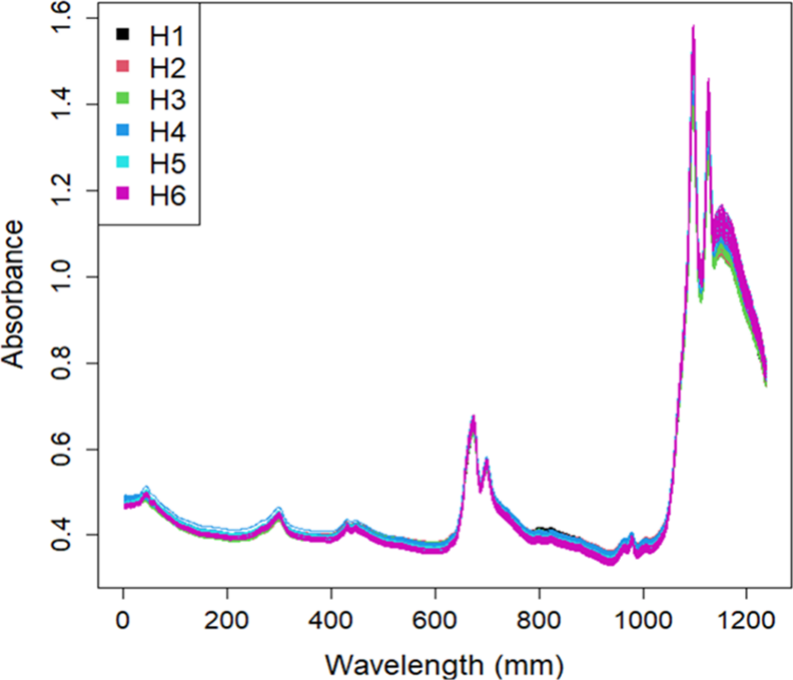
EVOO NIR spectra of harvests from H1 to H6.

Subsequently, the spectra underwent a smoothing process employing the Savitzky–Golay technique,^26^ effectively mitigating random noise within the instrumental signal. For each olive oil, 1237 pretreated NIR data points were obtained (representing energy absorbed at 1237 different wavelengths, from 800.62 to 2499.64 nm). Figure 1 shows a graphic depiction of these spectra, which includes data from harvests H1 through H6.

#### 2.1.2 Agro-Climatic Data

The study used agro-climatic data from the official website of the Andalusian Institute of Agricultural, Fisheries, Agrifood and Organic Production Research and Training. The website provides access to long-term information recorded by different AWSs with regular maintenance and thorough record reviews. Although there are ∼120 AWSs in all Andalusian provinces, the study only downloaded historical daily data from the 28 AWSs specified in Table 1 for the years 2005–2010. These AWSs were chosen for their proximity to the extraction points of the available oils. Table 2 includes the selected AWSs, their corresponding agro-climatic variables, and measurement units and procedures. Additionally, Table 2 presents the average values for the seven agro-climatic variables and each station. The substantial disparities in mean values across various AWSs make it logical to assign each EVOO the agro-climatic data linked to the closest AWS.

**Table 1:** Daily Agro-Climatic Information Downloaded from the Official Website

name	description	M.u.	measurement procedure
*Temp*	temperature	°C	a Pt1000 sensor measures temperature by detecting the change in the resistance of platinum based on the temperature.
*Hum*	relative humidity	%	relative humidity is measured using a solid-state capacitive device called HUMICAP 180. This device, made of a plastic polymer, absorbs humidity and changes its electrical characteristics, resulting in a decrease in electrical capacity.
*WSpe*	wind speed	m/s	a weathervane gauges wind speed by translating propeller rotation into an alternating current signal with a frequency that corresponds to the wind speed.
*WDir*	wind direction	°C	a weathervane measures the angle using a high-precision 10 KW conductive dimmer. The output signal is constant and proportional to the measured angle when a constant voltage is applied.
*Rad*	radiation	mj/m^2^	a pyrometer measures radiation using a silicon photoelectric cell sensitive to a wavelength range of 350 to 1100 nm. It is positioned to avoid shading from other sensors or tripod accessories.
*Precip*	precipitation	mm	the AWS uses a device with swinging containers to measure rainfall volume. Each contact with a tab (equivalent to 0.20 mm) represents the transfer of rainwater from one container to another.
*ETo*	evapotranspiration	mm/day	evapotranspiration (ET) refers to the loss of moisture (in mm per day) from a surface through direct evaporation and vegetation perspiration. ETP represents the maximum possible evaporation from fully vegetated soil under ideal conditions, assuming no water limitations. ETo is similar to ETP but is specific to standard crops such as cereals or alfalfa, with uniform height, active growth, full soil coverage, and no water deficit.

**Table 2:** Average Values for Each Agro-Climatic Measurement and AWS

province	station	code	*Temp (°C)*	*Hum (%)*	*WSpe(m/s)*	*WDir (deg)*	*Rad (MJ/m*^*2*^*)*	*Precip (mm)*	*ETo (mm/day)*
Cádiz	Villamartín	1	17.20	62.33	1.87	225.61	18.71	1.80	3.93
Córdoba	Adamuz	2	17.22	61.04	1.09	161.96	17.49	1.78	3.45
	Baena	3	17.43	57.45	1.14	189.63	18.56	1.38	3.59
	Belmez	4	15.56	60.52	2.12	223.16	18.03	1.48	3.88
	Cabra	5	16.71	56.81	0.92	157.20	17.50	1.91	3.17
	Cordova	6	17.37	61.90	1.66	174.70	17.84	1.83	3.87
	El Carpio	7	17.40	60.81	1.73	192.05	17.58	1.60	4.00
	Hinojosa del Duque	8	15.62	76.92	1.37	196.41	17.73	1.39	3.40
	Hornachuelos	9	17.93	57.62	1.28	174.87	18.05	1.81	3.67
	Palma del Río	10	17.87	59.82	1.73	166.78	18.44	1.79	4.06
	Santaella	11	17.66	58.04	1.81	180.87	17.87	1.63	4.00
Granada	Loja	12	16.12	58.40	1.82	188.18	18.31	1.25	3.97
	Pinos Puente	13	16.39	57.72	0.88	189.59	18.27	1.21	3.39
Jaén	Alcaudete	14	16.40	54.93	1.07	178.63	18.01	1.39	3.34
	Chiclana de Segura	15	15.48	60.37	1.40	219.46	16.99	1.49	3.52
	Jaén	16	16.48	58.80	1.00	178.27	18.35	1.45	3.46
	Higuera de Arjona	17	16.76	60.00	1.06	181.96	18.35	1.48	3.42
	Mancha Real	18	17.57	55.27	1.92	187.03	17.92	1.14	4.09
	Marmolejo	19	16.46	64.75	1.37	192.69	17.37	1.62	3.58
	Pozo Alcón	20	14.00	61.46	1.49	174.41	18.88	1.13	3.53
	San José de los Propios	21	16.72	54.21	2.00	217.76	19.20	1.10	4.21
	Santo Tomé	22	16.25	58.54	0.78	154.31	17.58	1.47	3.12
Málaga	Antequera	23	16.23	57.85	1.03	101.29	18.08	1.28	3.34
	Archidona	24	15.25	62.81	0.90	205.59	18.24	1.33	3.19
	Pizarra	25	18.03	61.79	1.50	135.19	18.21	1.43	3.80
	Sierra de Yeguas	26	15.97	63.51	2.26	204.31	18.42	1.36	3.91
Sevilla	Écija	27	17.62	60.21	1.59	157.96	18.42	1.57	3.90
	Osuna	28	17.36	59.87	2.30	175.12	18.77	1.34	4.40

### 2.2 Methodology

PCA is a dimensionality reduction technique that transforms a set of possibly correlated variables into a set of orthogonal variables known as *factors* or *principal components* to avoid multicollinearity. Therefore, this procedure is especially useful when the number of explanatory variables is much larger than the number of observations (such as in NIR spectral data). The process begins with standardizing the data, which ensures that each variable has a mean of zero and a variance of one by subtracting the mean and dividing by the standard deviation. Next, the covariance matrix of the standardized data (∑) is calculated. The eigenvalues of ∑ (λ) are calculated by solving the characteristic equation: det(∑ – λ*I*) = 0. Once the λ values are determined, the corresponding eigenvectors (ν) are determined by solving the equation: (∑ – λ*I*)*ν* = 0. The eigenvalues are ordered from largest to smallest, and the corresponding eigenvectors are selected to form the principal components matrix. Finally, the original data are transformed using this matrix, resulting in a reduced set of variables that retain most of the original variability. Typically, a few PCA components can explain a substantial portion of data variability and prevent overfitting. This method helps to simplify the data set while preserving its essential patterns. In addition to summarizing statistical information, PCA factors can serve as independent variables in subsequent statistical models to predict a qualitative or numerical dependent variable.

Furthermore, in statistical modeling, an input data set is partitioned into two subsets, representing about 75 and 25% of data. The first data subset (the *calibration* data) is used for fitting or training the model; the other subset (the *validation* data) permits the evaluation of the model predictions.

In the statistical classification, a qualitative, non-numeric variable is explained from a cause–effect model. In addition, the classification technique is known as *supervised* when the grouping into classes is previously known. Moreover, *machine learning* combines statistical modeling, artificial intelligence, and computer science in algorithms that mimic human learning. It automates analytical model building by learning from training data, solving tasks, and gradually improving accuracy over time. This study applies the following machine-learning-based supervised classification procedures for chemical data.^27,28^ First, the goal of LDA is to reduce the data dimensionality while preserving class-discriminatory information, facilitating better classification by identifying a linear combination of features that maximizes the separation between two or more classes. Second, *classification and regression trees* (CART) create a decision tree structure that predicts categorical outcomes in classification or continuous values in regression. The procedure recursively splits data into subsets based on feature values, aiming to minimize impurity in classification or variance in regression at each node, enabling accurate predictive modeling. Third, the goal of *k*-*nearest neighbor* (KNN) is to classify or predict data points based on their similarity to neighboring points. It accomplishes this by identifying the k-closest data points in the training set and assigning the majority class (for classification) or average values (for regression) to those neighbors to make predictions, with the goal of capturing local data trends. The goal of SVMs is to determine the optimal hyperplane for separating data into distinct classes while maximizing the margin (distance) between the classes. SVMs aim to create a decision boundary that best generalizes to unseen data, making it a powerful tool for classification and regression tasks. Finally, RFs improve the accuracy and robustness of decision trees by constructing an ensemble of multiple trees. It aims to reduce overfitting and enhance predictive performance by aggregating the results of many individual trees, making it a versatile and powerful machine-learning algorithm for classification and regression tasks. All these machine-learning algorithms were applied using the free software R.^29^ These procedures have associated *hyperparameters*: configuration settings that are external to the model, cannot be estimated from data, and must be specified by the practitioner before training the model. This study considers the default parameters of R, which were chosen based on general applicability and performance across a wide range of data sets. In particular, some of these hyperparameters were

- KNN: number of neighbors = 5, distance metric = “Euclidean.”
- SVN: kernel type = “radial,” coefficient = 0 (used in “poly” and “sigmoid” kernel), regularization parameter = 1 (controls the trade-off between achieving a low error on the training data and minimizing the norm of the weights).
- RF: number of trees = 500, maximum depth of the tree = none (nodes are expanded until all leaves are pure or contain less than the minimum samples split samples), minimum samples per leaf = 1 (minimum number of samples required to be at a leaf node).

All the aforementioned algorithms were assessed utilizing *accuracy* and *Kappa*. Accuracy is calculated as the ratio of correctly classified instances to the total. Kappa is a metric similar to classification precision, but it is standardized to account for random chance in the data set and is particularly useful for imbalanced category problems. Accuracy and Kappa were computed for calibration and validation, specifically considering the data utilized or not utilized in the fitting process. The arbitrary number seed was reset before each iteration to guarantee that all algorithms were evaluated by employing precisely the same subsets of calibration and validation (in addition to the same hyperparameters); thus, the outcomes can be comparable. In addition, a cross-validation procedure was used, which means that the described process of selecting calibration and validation sets was modified in different iterations (specifically, *n* = 25) to obtain more robust and independent results regardless of the considered random partition.

Moreover, the classification obtained using the PCA NIR factors was improved using agro-climatic information. Therefore, the agro-climatic data corresponding to the previous harvest and the nearest AWS (or the average of the nearest AWSs) were associated with each EVOO. A procedure was developed using R to select an agro-climatic variable and aggregate the daily measurements over a specific period. In particular, the function for assigning the agro-climatic information is the following
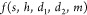
1with the following arguments:

- *s* represents the station (see the code of the nearest station in the first column of Table 2; this code was manually assigned),
- *h* is associated with the harvest, from H1 to H6,
- once the station and harvest are determined, a specific time frame is chosen (from *d*_1_ to *d*_2_) for aggregating daily agro-climatic measurements,
- *m* denotes the agro-climatic measurement: *Temp, Hum, WSpe, WDir, Rad, Precip*, and *ETo*.

Based on the selected period and meteorological criterion, the aggregated agro-climatic measurement is returned as a value.

To clarify the computational procedure of assigning data to each EVOO, an example is shown. The first EVOO of the sample is the following:

- *Characteristics*: harvest = H1, province = “Córdoba,” PDO = “Montoro–Adamuz,” variety = “Picual,” station = 20.
- *NIR spectra*: the original 1237 NIR spectrum data, which corresponds to the amount of energy absorbed at 1237 wavelengths, are summarized in 4 data points because the NIR spectral information is reduced to 4 PCA factors (as explained further below).
- *Agro*-*climatic information*: Function (1), in this case, is the following: *f*(20,H1,*d*_1_,*d*_2_,*m*). Daily data for 2005, before H1, can be accumulated from 1 day (*d*_1_) to another (*d*_2_) (in particular, data are aggregated monthly) and for the different agro-climatic measurements: *Temp, Hum, WSpe, WDir, Rad, Precip*, and *ETo*. Therefore, a 7 × 12 matrix with the following format was associated with this first EVOO:

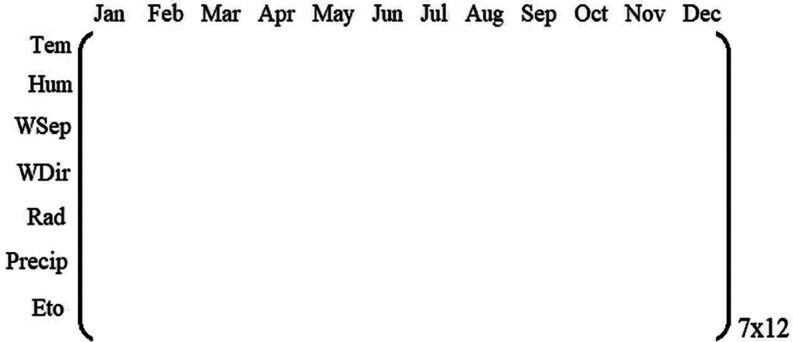


These matrices were included in a list of R to be indexed in the following analyses. Finally, regarding the specific R software packages, “fda.usc,”^30^ “pls,”^31^ and “caret”^32^ were used to represent the spectral derivatives, obtain the NIR spectral PCA factors, and apply machine-learning classification, respectively.^33^

## 3 Results

### 3.1 Analysis of NIR Spectra and Agro-Climatic Data

An NIR spectrum displays absorbance, which represents the amount of energy absorbed by an EVOO at each wavelength. This data set can be treated as continuous (i.e., a curve) instead of an extensive discretized data set. This fact permits us to obtain its derivatives, which, along with the original spectra, contain valuable information for the qualitative and quantitative characterization of oils.

**Figure 2:**
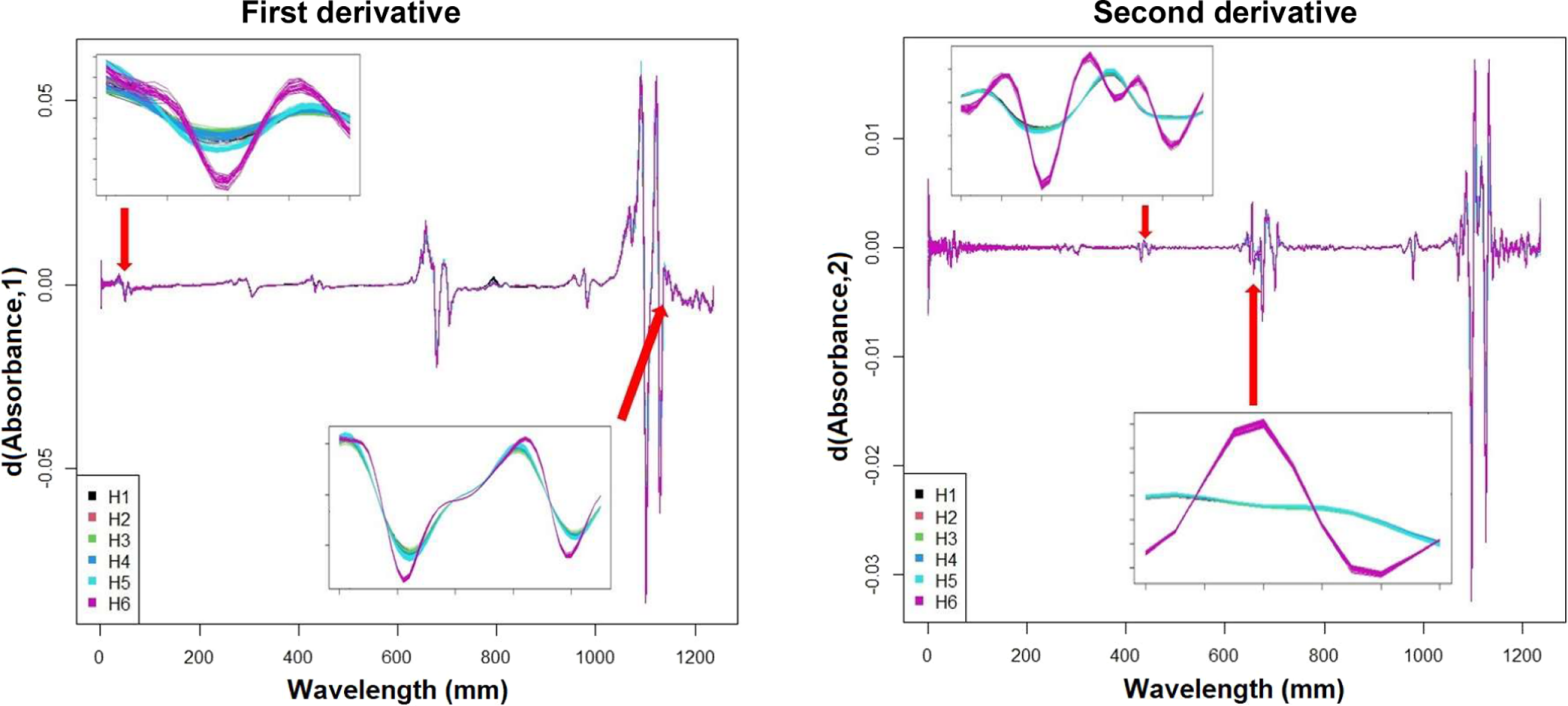
First and second derivatives of the NIR spectra and magnification of the maximum discrepancies.

Figure 1 shows the NIR spectra and Figure 2 shows the first and second derivatives. Figure 2 also shows some of the maximum discrepancies that exist in the evolution of the derivative spectra, which correspond to the spectra associated with the last harvest (H6, in pink).

**Figure 3:**
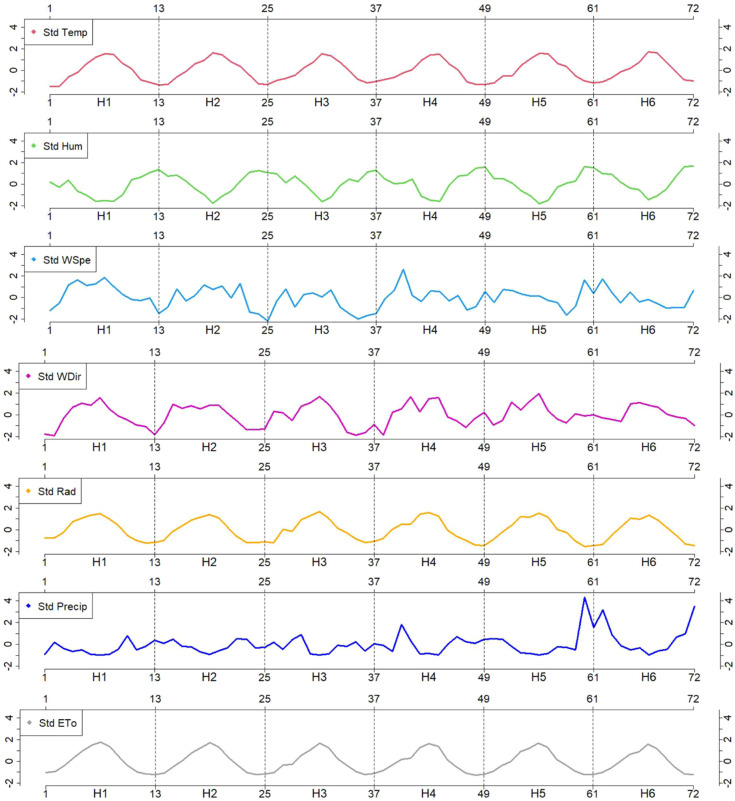
Evolution of the agro-climatic measurements during the six harvests, accumulated monthly and standardized.

**Figure 4:**
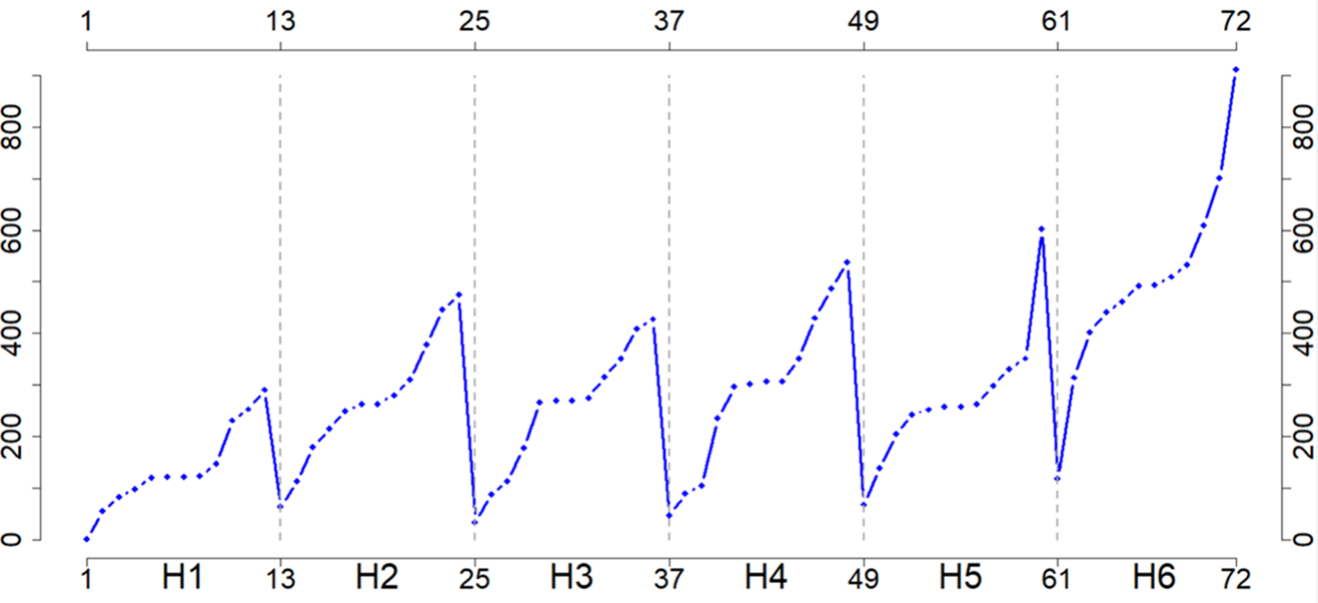
Monthly cumulative precipitation (mm) for every harvest.

Daily values for the seven considered agro-climatic measurements (*Temp, Hum, WSpe, WDir, Rad, Precip*, and *ETo*) were accumulated monthly and standardized to remove the influence of the measurement units. These dimensionless values (Figure 3) exhibited a cyclic pattern across most variables. Specifically, *Temp, WSpe, WDir, Rad*, and *ETo* displayed similar trends, whereas *Hum* and *Precip* showed opposite tendencies. Notably, *Precip* (in blue) showed exceptionally high values toward the end of H5 and at the beginning and end of H6. This irregular behavior is further evident in Figure 4, which illustrates the accumulated mean monthly values.

Therefore, the anomalous precipitation and derivative NIR spectra from H6 raise the question of how precipitation, in particular, and agro-climatic conditions, in general, affects the NIR spectra. Moreover, could agro-climatic information provide valuable information to improve quantitative or qualitative estimations of EVOO, making it beneficial for authentication? In response to these questions, our research group developed the following studies. First, one study^25^ used redundancy analysis to highlight the linear relationships between agro-climatic data and NIR spectra; second, some studies showed how incorporating agro-climatic information into NIR spectra could improve fatty acid profile estimation, whether using a discrete^31^ or continuous^24^ approach. Furthermore, the purpose of this study was to determine if agro-climatic data could be added to NIR spectral data to improve EVOO classification, specifically to predict its geographical origin or PDO.

### 3.2 Classification from Spectral and Agro-Climatic Information

#### 3.2.1 Spectral Information Alone

The large quantity of NIR spectral information was summarized using the PCA analysis. Considering recommendations in the literature to prevent overfitting (as the usual Kaiser Criterion), the number of retained PCA factors was four: the percentages of variance for the first four factors (PCA1, PCA2, PCA3, and PCA4) were 70.25, 25.75, 1.76, and 1.31% (99.07% in total), respectively, with the eigenvalue associated with PCA5 of <1. In addition, as demonstrated, increasing the retained factors does not improve the capability of the subsequent classification procedures.

Afterward, these PCA factors were included as independent variables in the classification models that first considered the variable province (“Cádiz,” “Córdoba,” “Granada,” “Jaén,” “Málaga,” or “Sevilla”) and then the variable PDO (‘Antequera,” “Campiña de Jaén,” “Estepa,” “Lucena,” “Montoro–Adamuz,” “Poniente de Granada,” “Priego Córdoba,” and “Sierra Cádiz”) as dependent variables (Y). The results using only spectral information as explanatory variables are as follows. Table 5 and Figure 6 show that for the province, RF (53.9473%) and LDA (35.4101%) exhibited the best calibration classifications for accuracy and Kappa, respectively. As shown in Table 5 and Figure 7, LDA provided the best percentages (62.9630% and 50.4587%) of correct validation classification for accuracy and Kappa, respectively. In the case of PDO (Table 6 and Figure 8), the best calibration classification percentages were provided by RF (56.0680%) and LDA (47.9228%) for accuracy and Kappa, respectively. As shown in Table 6 and Figure 9, LDA provided the best validation classification percentages of 40.7407% and 31.7570% of correct classification for accuracy and Kappa, respectively.

#### 3.2.2 Spectral plus Agro-Climatic Information

The objective of this section was to improve the classification (calibration and validation) percentages for accuracy and Kappa obtained based on the province and PDO. With this goal, agro-climatic information was assigned to each EVOO, which was added to the spectral information among the explanatory variables of the models.

**Figure 5:**
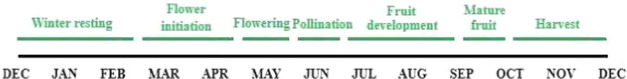
Phenological phases of olives throughout a year.

In particular, Function 1, described in the methodology, was applied considering the corresponding EVOO, AWS, and harvest. The downloaded seven agro-climatic measurements (*Temp, Hum, WSpe, WDir, Rad, Precip*, and *ETo*) were accumulated monthly, providing a list of 12 matrices of dimensions 222 × 7 from January to December. The monthly aggregation of the agro-climatic measurement was made to adequately relate these values to the phenological cycle of the olive groove. Figure 5 shows that this cycle was not uniformly distributed; thus, the effect could be independently analyzed. Other authors^34^ have also studied the influence of meteorological data on Italian oil production by considering monthly meteorological variables. Another study^35^ highlighted the influence of agro-climatic conditions on fruit set, fruit size, fruit yield, and oil content of olive cultivars, as well as the phenological phase of olive fruit development.

Subsequently, the information provided by these monthly aggregated agro-climatic variables was added to the PCA spectral information in the classification models. The results were analyzed by accuracy and Kappa when province and PDO were considered as dependent variables. The hyperparameters of the classification procedures were the same and the arbitrary number seed was reinitialized before each iteration to guarantee that the process utilized identical calibration and validation subsets, thus enabling direct comparability of the outcomes (even when only PCA spectral information was incorporated). Moreover, a cross-validation procedure was programmed to select the calibration and validation subsets across 25 iterations to increase the level of robustness and independence of the results.

**Figure 6:**
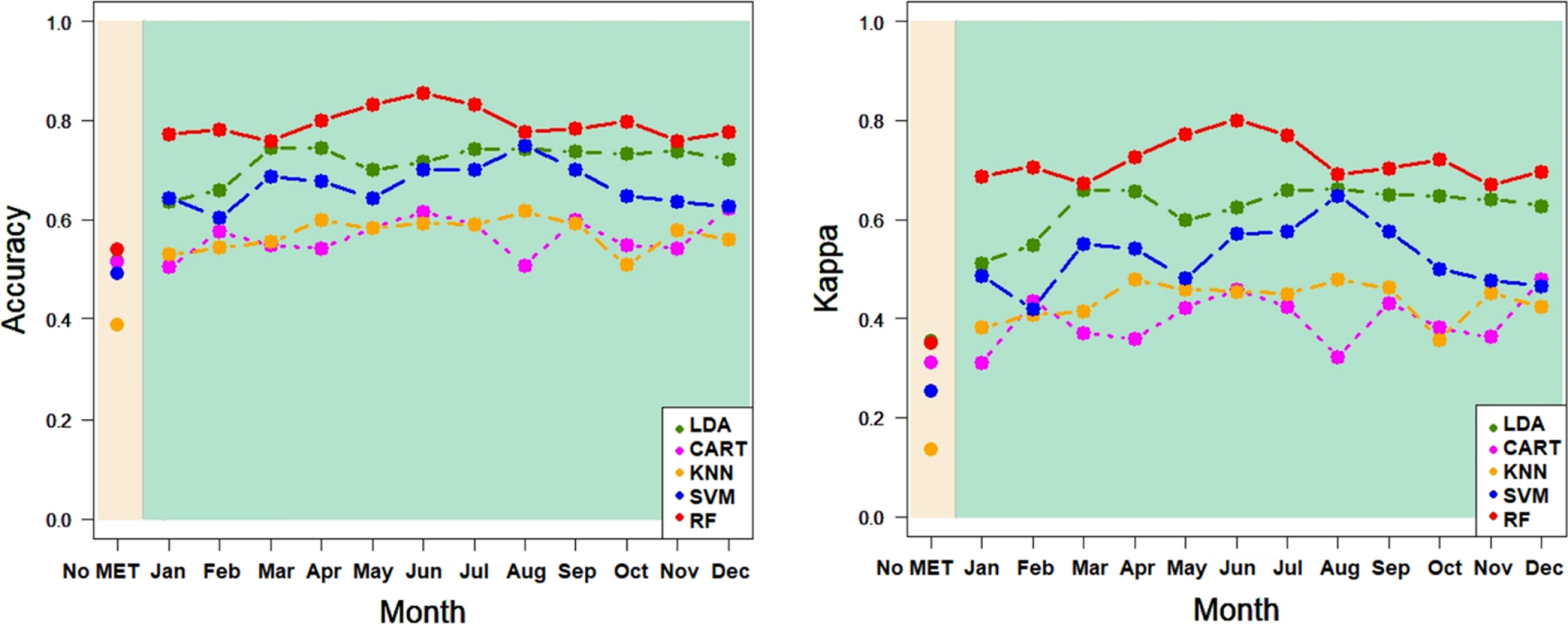
Classification ratios in the province for LDA, CART, KNN, SVM, and RF: accuracy and Kappa for CALIBRATION^(*)^ In beige, ratios for models without agro-climatic information; in green, ratios for models with agro-climatic information, aggregated for each month. KNN, SVM, and RF use the default hyperparameters of R indicated in Section 2.2.

**Figure 7:**
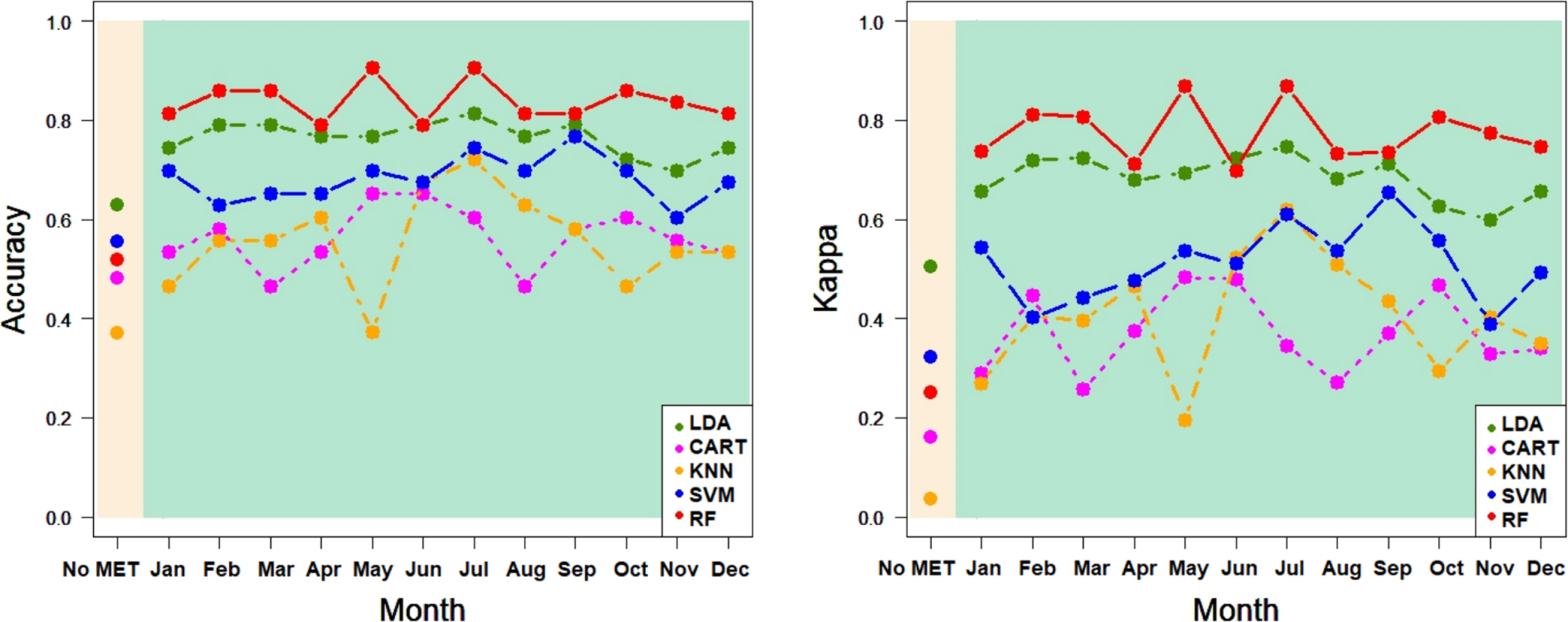
Ratios of classification in the province for LDA, CART, KNN, SVM, and RF: accuracy and Kappa for VALIDATION^(*)^ In beige, ratios for models without agro-climatic information; in green, ratios for models with agro-climatic information, aggregated for each month. KNN, SVM, and RF use the default hyperparameters of R indicated in Section 2.2.

**Figure 8:**
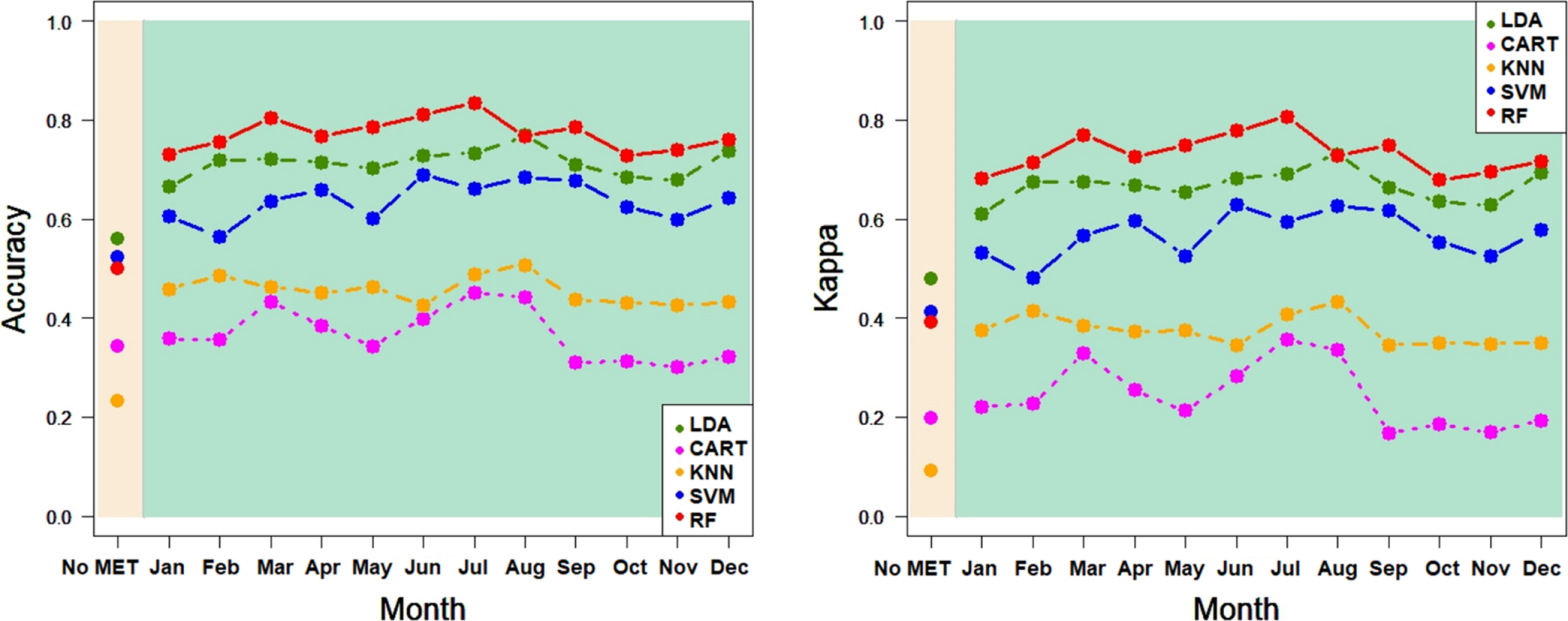
Ratios of classification in PDO for LDA, CART, KNN, SVM, and RF: accuracy and Kappa for CALIBRATION^(*)^ In beige, ratios for models without agro-climatic information; in green, ratios for models with agro-climatic information, aggregated for each month. KNN, SVM, and RF use the default hyperparameters of R indicated in Section 2.2.

**Figure 9:**
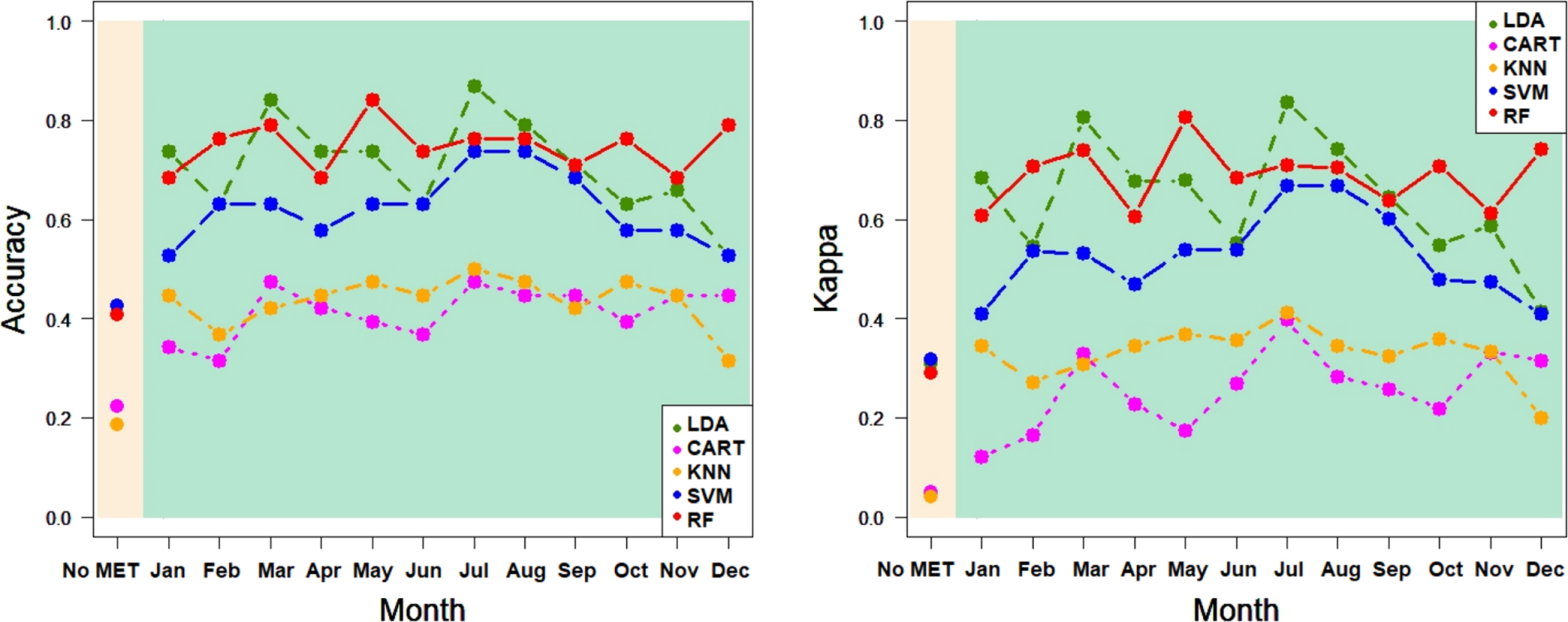
Classification ratios in PDO for LDA, CART, KNN, SVM, and RF: accuracy and Kappa for VALIDATION^(*)^ In beige, ratios for models without agro-climatic information; in green, ratios for models with agro-climatic information, aggregated for each month. KNN, SVM, and RF use the default hyperparameters of R indicated in Section 2.2.

Figures 6–9 show the correct classification ratios for each machine-learning procedure, highlighting that the LDA, CART, KNN, SVM, and RF-classification models improved the calibration and validation for province and PDO, for accuracy and Kappa measures, when agro-climatic information was added to NIR spectral information. However, the improvement varied depending on the aggregation month of the agro-climatic measurements. In general, RF (red) and LDA (green) provided the best results when agro-climatic information from June and July (the stages of pollination and the beginning of the olive fruit development) was included in the statistical model. In addition, Tables 3 and 4 show the percentages of improvement for province and PDO, for calibration and validation and each machine-learning procedure. This percentage of improvement was calculated as 100(Final% – Initial%)/Initial%, where Initial% and Final% represent the % of correct classification without and with agro-climatic information, respectively. Therefore, a great improvement for province was obtained using KNN (59.0264 and 252.7475% for accuracy and Kappa, respectively, for calibration and 94.6512% and 130.0498% for accuracy and Kappa, respectively, for validation). Moreover, for PDO, KNN showed great improvement for calibration accuracy and Kappa (117.5867 and 372.5260%, respectively) and validation accuracy (170.000%), but CART showed the highest validation Kappa value (225.6687%).

**Table 3:** Percentage of Improvement^a^ for Province for Each Classification Procedure

	LDA	CART	KNN	SVM	RF
	calibration				
accuracy	44.0096	21.1141	59.0264	53.0264	58.6694
Kappa	86.5730	53.7512	252.7475	157.3955	128.5144
	validation				
accuracy	29.2750	35.2415	94.6512	38.1395	74.9169
Kappa	13.7645	66.3653	130.0498	20.8082	17.7686

a Improvement% = 100(Final% – Initial%)/Initial%, where Initial% and Final% represent % correct classification without and with agro-climatic information, respectively.

**Table 4:** Percentage of Improvement^a^ for PDO for Each Classification Procedure

	LDA	CART	KNN	SVM	RF
	calibration				
accuracy	37.0944	31.5504	117.5867	31.6819	66.7117
Kappa	52.6445	81.2014	372.5260	52.9712	105.9565
	validation				
accuracy	113.1579	113.1579	170.0000	72.9977	106.6986
Kappa	22.38635	225.6687	19.68196	63.5071	32.5861

a Improvement% = 100(Final% – Initial%)/Initial%, where Initial% and Final% represent % correct classification without and with agro-climatic information, respectively.

More specifically, as shown in Table 5 for provinces and accuracy, RF provided the best percentage of correct classification in calibration (53.9347%) from only the spectral information (beige part of figure). This percentage increased to 85.5780% when agro-climatic information (from June) was included in the same classification model (green part of figure). With respect to the validation, LDA correctly classified 62.9630% of the cases, and when the agro-climatic information (aggregated for July) was added, RF provided a correct EVOO classification of 90.6977%. The calibration and validation percentages obtained for Kappa were slightly lower but showed the same tendency and even higher differences in the percentages of correct classification (44.6802 and 36.4315%, respectively). Overall, the elevated level of improvement was emphasized by the corresponding percentages of improvement for the calibration accuracy and Kappa and the validation accuracy and Kappa (last row of Table 5): 58.6697, 126.1793, 44.0492, and 72.2006%, respectively.

**Table 5:** Best Procedures for Correct Classification of Province

		calibration		validation	
		accuracy	Kappa	accuracy	Kappa
without AGRO-CLIM	best procedure	RF	LDA	LDA	LDA
	correct %	53.9347	35.4101	62.9630	50.4587
with AGRO-CLIM	best procedure	RF	RF	RF	RF
	month	June	June	July	June
	correct %	85.5780	80.0903	90.6977	86.8902
	**difference** %	31.6433	44.6802	27.7347	36.4315
	ai**mprovement** %	**58.6697**	**126.1793**	**44.0492**	**72.2006**

a Improvement% = 100(Final% – Initial%)/Initial%.

Table 6 shows the PDO classification results: LDA provided a correct (accuracy) classification for a calibration of 56.0680% with the percentage increasing to 83.4510% when agro-climatic (aggregated for July) information was included in the RF model. With respect to validation, LDA classified only 40.7407% of EVOO, whereas the percentage increased to 86.8421% for RF with agro-climatic information from July. Finally, as in the case of the province, the calibration and validation percentages obtained for Kappa were slightly lower, but the differences in the percentages of the correct classification were higher (32.7738% and 51.9059%, respectively). Therefore, the highest percentages of improvement for the calibration accuracy and Kappa and the validation accuracy and Kappa (the last row of Table 6) were 48.8389%, 68.3887%, 113.1581%, and 163.4471%, respectively.

**Table 6:** Best Procedures of Correct Classification for PDO

		calibration		validation	
		accuracy	Kappa	accuracy	Kappa
without AGRO-CLIM	best procedure	RF	LDA	LDA	LDA
	correct %	56.0680	47.9228	40.7407	31.7570
with AGRO-CLIM	best procedure	RF	RF	RF	RF
	month	June	June	July	June
	correct %	83.4510	80.6966	86.8421	83.6629
	**difference** %	27.3830	32.7738	46.1014	51.9059
	a**improvement** %	**48.8389**	**68.3887**	**113.1581**	**163.4471**

a Improvement% = 100(Final% – Initial%)/Initial%.

**Figure 10:**
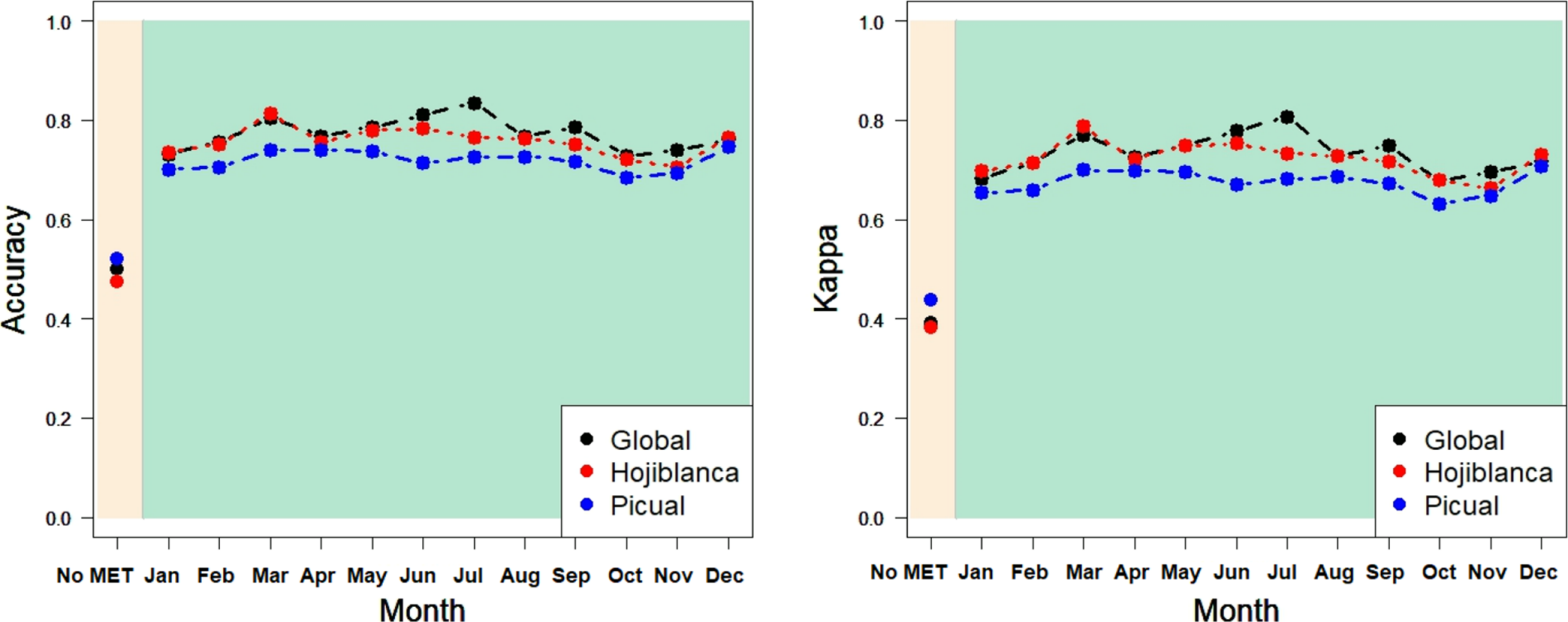
Ratios of RF-classification in PDO for global oils and “Hojiblanca” and “Picual” varieties: accuracy and Kappa for CALIBRATION^(*)^ In beige, the ratios for models without agro-climatic information; in green, the ratios for models with agro-climatic information, aggregated for each month. KNN, SVM, and RF use the default hyperparameters of R indicated in Section 2.2.

To analyze the robustness and generalizability of the PDO classification findings, Figure 10 shows the RF results (the procedure providing the best results in general), comparing the ratios for the global EVOO sample and the oil subsamples from the “Hojiblanca” and “Picual” olive varieties. Notably, these two olive varieties were the most prevalent among the studied PDO, representing 29.8 and 34.7%, respectively, of the total oils. The calibration results, which were cross-validated using 25 different calibration and validation subsets, again showed that adding agro-climatic information to the NIR spectral data improved PDO classification. Furthermore, the classification percentages were quite similar for the global sample and the subsamples of the “Hojiblanca” and “Picual” varieties (with the latter showing a slightly lower accuracy and Kappa rate).

**Figure 11:**
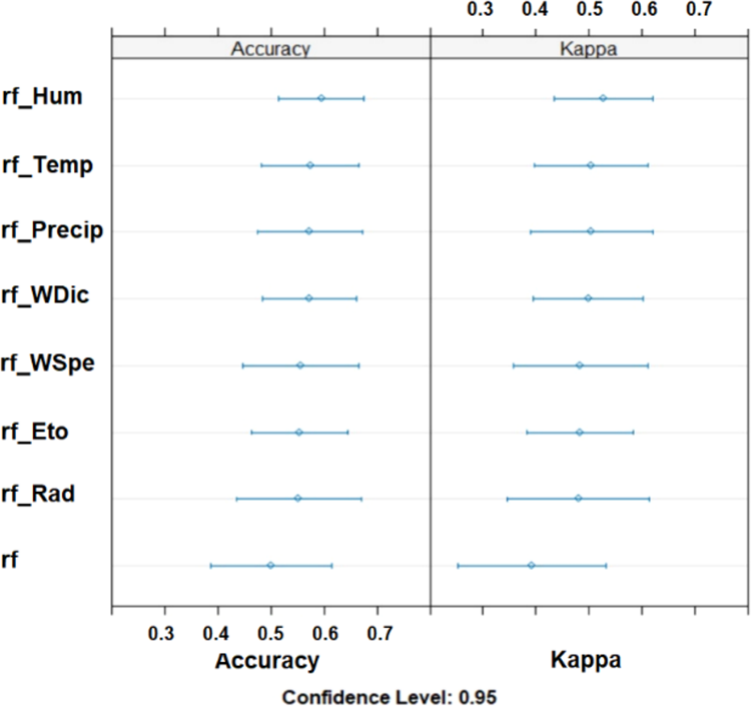
Confidence intervals for the ratios of RF-classification in PDO for models without and with agro-climatic information, introduced one variable at a time: accuracy and Kappa for CALIBRATION.

Finally, we wanted to identify the specific agro-climatic parameters that were most influential in improving classification performance. Figure 11 shows the confidence intervals (to 95%) for the PDO classification ratios from RF without and with agro-climatic information (*Hum*, *Temp*, *Precip*, *WDic*, *Wsped*, *ETo*, and *Rad*) introduced one variable at a time. The greatest improvement in classification (accuracy and Kappa) occurred when the humidity information (*Hum*) was introduced into the model. Nevertheless, the introduction of any agro-climatic variable led to an improvement in classification.

## 4 Discussion

In this study, PCA was used to condense the high-dimensional information on EVOO NIR spectra into a few components that collectively captured ∼100% of the data variability. Subsequently, these PCA components were integrated as independent variables in various machine-learning classification models (including LDA, CART, KNN, SVM, and RF) to forecast the geographical origin (province) and PDO of EVOO. Subsequently, agro-climatic information downloaded from the official website of the Andalusian AWSs was added to the previous models. The calibration and validation results were evaluated using accuracy and Kappa values (using the same number of seeds and hyperparameters to obtain comparable outcomes) for both classification variables. In addition, the results were cross-validated by changing the calibration and validation sets in 25 iterations and assigning an average value to the previous evaluation measures. These results highlight the improvement for each classification procedure when agro-climatic information is added to the NIR spectral data. However, although this improvement was evident for all phenological stage months of olives for which agro-climatic information was aggregated and processed, it was higher for specific months and classification techniques. Thus, RF and LDA provide the best calibration and validation results (based on accuracy and Kappa) for the province and PDO when agro-climatic information is added to NIR spectral data, preferably when this agro-climatic information is associated with the phenological stage corresponding to olive fruit development rather than winter resting stages, when the fruit is absent in the olive tree. Nevertheless, the calibration and validation improvement percentages for the province and PDO can be emphasized because they range from 44.0492% to 163.4471%. In particular, the humidity (*Hum*) variable provides the greatest improvement in PDO classification in calibration. In addition, to analyze the robustness of the outcomes in the PDO classification, the RF calibration results (the best procedure for PDO) of the global EVOO sample were compared to the oils from “Hojiblanca” and “Picual,” the most representative olive varieties. The cross-validated correct PDO classification ratios were quite similar when they were calculated for a specific variety. Other olive varieties included in this study (other than “Hojiblanca” and “Picual”) were not analyzed separately because of the small sample size of the corresponding subgroups, which represents a limitation of this study. Future research will focus on the period when a greater improvement is obtained (approximately March to July) and examine including MIR or Raman spectral information into the agro-climatic data.

## Data Availability

The agro-climatic data can be freely downloaded from the official website of the “Andalusian Institute of Agricultural, Fisheries, Agrifood and Organic Production Research and Training”: https://www.juntadeandalucia.es/agriculturaypesca/ifapa/riaweb/web/datosabiertos.

## Supporting Information Available

The R code and the data supporting the analyses (ZIP)
Frequency distribution for harvest, province, PDOs, and olive varieties (Table A.1) (PDF)
