# Supplementary material for: Agro-Climatic Information to Enhance the Machine-Learning Classification of Olive Oils from Near-Infrared Spectra
Source: ACS Agric Sci Technol. 2024 Oct 11;4(11):1194–205. doi: 10.1021/acsagscitech.4c00355 (PMC11578292; doi:10.1021/acsagscitech.4c00355)
Supplement: Supplementary file 2 — as4c00355_si_002.pdf [file as4c00355_si_002.pdf]

Supporting information to the manuscript:

Agro-climatic Information to Enhance the Machine-Learning Classification of Olive Oils From Near-Infrared Spectra

María Isabel Sánchez-Rodríguez*[1], Elena Sánchez-López[2], Alberto Marinas[3],

José María Caridad[4] and Francisco José Urbano[5]

∗Corresponding author: td1sarom@uco.es.

Affiliations:

[1]Department of Statistics and Business. University of Cordoba. Faculty of Law and Business. Avda. Puerta Nueva, s/n. 14071. Cordoba. Spain. ORCID: 0000-0003-0657-7823; email: td1sarom@uco.es.

[2]Department of Organic Chemistry. University of Cordoba. Campus de Rabanales, Marie Curie Building. 14014. Cordoba. Spain. ORCID: 0000-0002-4885-6065; email: g02saloe@uco.es.

[3]Department of Organic Chemistry. University of Cordoba. Campus de Rabanales, Marie Curie Building 14014. Cordoba. Spain. ORCID: 0000-0002-2693-0711; email: alberto.marinas@uco.es.

[4]Department of Statistics and Business. University of Cordoba. Faculty of Law and Business. Avda. Puerta Nueva, s/n. 14071. Cordoba. Spain. ORCID: 0000-0003-4558-6618; email: ccjm@uco.es.

[5]Department of Organic Chemistry. University of Cordoba. Campus de Rabanales, Marie Curie Building. 14014. Cordoba. Spain. ORCID: 0000-0002-3489-1601; email: qo1urnaf@uco.es.

Supplementary Information:

Table A.1. *Frequency distribution for harvest, province, PDOs, and olive varieties*

Variable	Frequency
**HARVEST**	
H1 (2005–06)	36
H2 (2006–07)	31
H3 (2007–08)	43
H4 (2008–09)	42
H5 (2009–10)	40
H6 (2010–11)	30
PROVINCE	
Cádiz	25
Córdoba	64
Granada	25
Jaén	36
Málaga	44
Sevilla	28
PDO	
Antequera	41
Campiña de Jaén	21
Estepa	22
Lucena	25
Montoro–Adamuz	45
Poniente de Granada	24
Priego de Córdoba	23
Sierra de Cádiz	21
VARIETY	
Hojiblanca	65
Picual	77
Other (arbequina, manzanilla…)	80
